# *In silico* Guided Drug Repurposing: Discovery of New Competitive and Non-competitive Inhibitors of Falcipain-2

**DOI:** 10.3389/fchem.2019.00534

**Published:** 2019-08-06

**Authors:** Lucas N. Alberca, Sara R. Chuguransky, Cora L. Álvarez, Alan Talevi, Emir Salas-Sarduy

**Affiliations:** ^1^Laboratory of Bioactive Compounds Research and Development (LIDeB), Department of Biological Sciences, Exact Sciences College, Universidad Nacional de La Plata, La Plata, Argentina; ^2^Departamento de Biodiversidad y Biología Experimental, Facultad de Farmacia y Bioquímica, Facultad de Ciencias Exactas y Naturales, Consejo Nacional de Investigaciones Científicas y Técnicas, Instituto de Química y Fisico-Química Biológicas (IQUIFIB) “Prof. Alejandro C. Paladini”, Universidad de Buenos Aires, Buenos Aires, Argentina; ^3^Instituto de Investigaciones Biotecnológicas “Dr. Rodolfo Ugalde”, Universidad Nacional de San Martín, CONICET, Buenos Aires, Argentina

**Keywords:** malaria, *Plasmodium falciparum*, falcipain-2, drug repositioning, virtual screening, drug rescue, odanacatib, methacycline

## Abstract

Malaria is among the leading causes of death worldwide. The emergence of *Plasmodium falciparum* resistant strains with reduced sensitivity to the first line combination therapy and suboptimal responses to insecticides used for Anopheles vector management have led to renewed interest in novel therapeutic options. Here, we report the development and validation of an ensemble of ligand-based computational models capable of identifying falcipain-2 inhibitors, and their subsequent application in the virtual screening of DrugBank and Sweetlead libraries. Among four hits submitted to enzymatic assays, two (odanacatib, an abandoned investigational treatment for osteoporosis and bone metastasis, and the antibiotic methacycline) confirmed inhibitory effects on falcipain-2, with Ki of 98.2 nM and 84.4 μM. Interestingly, Methacycline proved to be a non-competitive inhibitor (α = 1.42) of falcipain-2. The effects of both hits on falcipain-2 hemoglobinase activity and on the development of *P. falciparum* were also studied.

## Introduction

Despite decades of successful interventions aimed at reducing its incidence and mortality, malaria continues being one of global leading causes of death, being the main global cause globally in the 5- to 14-year-old population and the third cause among children below five (World Health Organization (WHO), 2017; Ritchie and Roser, 2018). The most recent estimates from the World Health Organization (WHO) report around 216 million cases and 445,000 related deaths worldwide in 2016 (Ritchie and Roser, 2018). The emergence of *Plasmodium falciparum* drug-resistant strains with reduced sensitivity to the first line artemisinin combination therapy and suboptimal response to insecticides used for vector management pose a threat to control interventions (Satimai et al., 2012; Ajayi and Ukwaja, 2013; Kisinza et al., 2017). Accordingly, novel therapeutic options are urgently required.

Falcipains are *P. falciparum* cysteine proteases involved in different processes of the erythrocytic cycle of the parasite, including hydrolysis of host hemoglobin and erythrocyte invasion and rupture. Four falcipains have so far been identified, with falcipain-2 and falcipain-3 constituting promising targets in the search for novel therapies due to their significant hemoglobinase capacity (Marco and Coterón, 2012; Bekono et al., 2018).

Drug repurposing involves finding novel medical uses for existing drugs, including approved, investigational, discontinued, and shelved therapeutics. Repurposing a drug has several advantages in comparison to *de novo* drug discovery, since the new therapeutic indication is built on already available pharmacokinetic, toxicological, and manufacturing data, thus leading to therapeutic solutions in a expedite manner (Ferreira and Andricopulo, 2016; Corsello et al., 2017). While the best-known examples of successful repurposing have been serendipitous or arose from intelligent exploitation of side effects (Corsello et al., 2017; Talevi, 2018), the drug discovery community has recently focused on systematic, large-scale repurposing efforts, including the use of genomic tools, and *in silico* and *high-throughput* screening (Jin and Wong, 2015; Talevi, 2018; Yella et al., 2018). The Virtual lock-and-key approach (Lauria et al., 2011, 2014; Tutone et al., 2017) and the BIOlogical Target Assignation method (Lauria et al., 2014) can be mentioned among many other interesting examples of the use of computational resources to deepen the rational basis of drug repurposing programs.

Here, we have implemented a computer-aided drug repurposing campaign to discover new inhibitors of falcipain-2. Four hits were acquired and tested against the enzyme, with two of them confirming inhibitory activity. The abandoned drug odanacatib displayed competitive inhibition, while the antibiotic methacycline also showed inhibitory effects through non-competitive inhibition.

## Materials and Methods

### Dataset Collection

**Figure 1:**
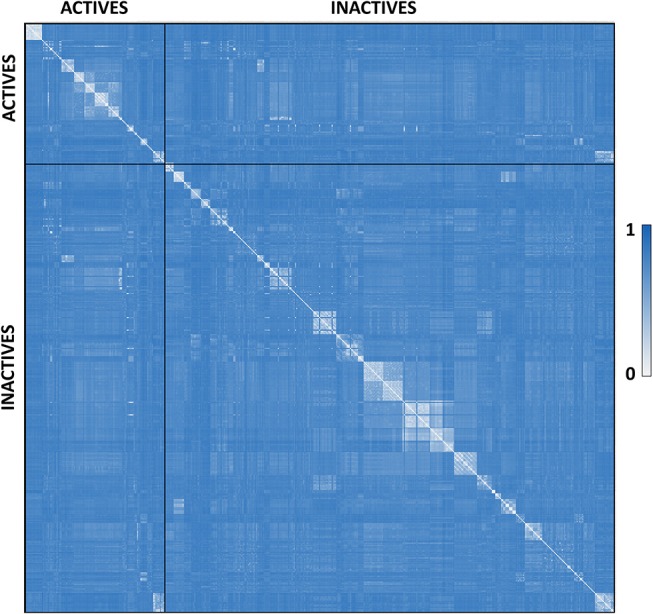
Dissimilarity heatmap of the whole dataset. Light areas indicate high similarity between the compared compounds while dark blue areas indicate low similarity between the compared compounds.

*P. falciparum* falcipain-2 inhibitors were compiled from literature. A total of 515 compounds previously assayed against falcipain-2 were collected from over 20 original articles, conforming the dataset used for model calibration and validation (Domínguez et al., 1997; Chiyanzu et al., 2003; Shenai et al., 2003; Desai et al., 2004, 2006; Greenbaum et al., 2004; Fujii et al., 2005; Goud et al., 2005; Micale et al., 2006; Valente et al., 2006; Biot et al., 2007; Chipeleme et al., 2007; Li et al., 2009; Hans et al., 2010; Praveen Kumar et al., 2011; Shah et al., 2011; Huang et al., 2012; Luo et al., 2012; Conroy et al., 2014; Ettari et al., 2014; Jin et al., 2014; Wang et al., 2014; Weldon et al., 2014; Bertoldo et al., 2015; Mundra and Radhakrishnan, 2015a,b; Sharma et al., 2015; Singh et al., 2015; Schmidt et al., 2016). Such compounds were labeled as ACTIVE or INACTIVE according to their reported inhibitory data. The ACTIVE category included compounds with IC_50_ ≤ 5 μM, plus compounds with a percentage of inhibition ≥ 50% against the enzyme at 10 μM or ≥ 80% at 20 μM (when a single-point inhibition assay was reported). When none of the previous conditions were met, the compound was labeled as INACTIVE. Considering such criteria, the dataset includes 122 active compounds and 393 inactive compounds. Such dataset was curated using the standardization tool available in Instant JCHEM v. 17.2.6.0 (Chemaxon). The molecular diversity of the whole dataset and within each category can be appreciated in the heatmap displayed in Figure 1, which shows, for every compound pair, the Tanimoto distance computed using ECFP_4 molecular fingerprints. The heatmap was built using Gitools v. 2.3.1 (Perez-Llamas and Lopez-Bigas, 2011) and Tanimoto distances were calculated using ScreenMD—Molecular Descriptor Screening v. 5.5.0.1 (ChemAxon). The dataset is included as Supplementary Data Sheet 1.

### Dataset Partition

It has been observed that rational/representative splitting of datasets into training and test sets tends to produce models with better predictivity (Golbraikh et al., 2003; Leonard and Roy, 2006; Martin et al., 2012). In the present study, a representative sampling procedure was thus used to divide the datasets into: (a) a training set, that was used to calibrate the models and; (b) a test set, that was used to independently assess model predictivity. Such representative partition of the dataset resulted from a serial combination of two clustering procedures. First, we have used the hierarchical clustering method included in LibraryMCS software (version 17.2.13.0–ChemAxon), which relies on the Maximum Common Substructure (MCS). A compound from each of the resulting cluster was randomly chosen and used as a seed to perform a non-hierarchical clustering using the k-means algorithm, as implemented in Statistica 10 Cluster Analysis module (Statsoft). Hierarchical clustering allowed deciding on an initial partition of n molecules into k groups, and this preliminary clustering was then optimized through the non-hierarchical procedure, as suggested by Everitt et al. (2011). We have previously used this combined approach for representative dataset partitioning, with good results (Alberca et al., 2016, 2018; Gantner et al., 2017). The clustering procedure was performed separately for the ACTIVE and INACTIVE categories.

75% of the compounds in each cluster of the ACTIVE category were kept for the training set (making a total of 91 compounds); an equal number of compounds were taken from the INACTIVE category clusters (23% of each INACTIVE cluster). We have under-sampled the INACTIVE category, so that a balanced training sample (comprising an identical proportion of active and inactive examples) was obtained and model bias toward predicting the larger category was avoided. The remaining 31 active and 302 inactive compounds were assigned to test set (333 compounds in total), which was later used for external validation of the models.

### Molecular Descriptor Calculation and Modeling Procedure

3,668 conformation-independent descriptors were computed with Dragon 6.0 software. A random subspace-based method was applied to obtain 1,000 descriptor subsets of 200 potential independent variables each. In the random subspace approach, the molecular descriptors are randomly sampled, and each model is trained on one subset of the feature space (Yu et al., 2012; El Habib Daho and Chikh, 2015); as a result, individual models do not over-focus on features that display high explanatory power in the training set.

A dummy variable (class label) was used as dependent variable. It was assigned observed values of 1 for compounds within the ACTIVE category and observed values of 0 for compounds in the INACTIVE one. Using a Forward Stepwise procedure and a semi-correlation approach (Toropova and Toropov, 2017), 1,000 linear classifiers were obtained, one from each of the random subsets of features. In order to avoid overfitting, only one molecular descriptor every 12 training instances was allowed into each model, with no more than 12 independent variables per model. Also, a maximal Variance Inflation Factor of 2 was tolerated. No descriptor with regression coefficient with *p*-value above 0.05 was allowed into the model. R language and environment was used for all data analysis. The R package data table (https://cran.r-project.org/package=data.table) was used to handle datasets.

The robustness and predictive ability of the models were initially estimated through randomization and Leave-Group-Out cross-validation tests. In the case of randomization, the class label was randomized across the compounds in the training set. The training set with the randomized dependent variable was then used to train new models from the descriptor selection step. Such procedure was repeated 10 times within each descriptor subset and the 95% confidence interval was built around the mean accuracy of the randomized models. It is expected that the randomized models will perform poorly compared to the real ones. Regarding the Leave-Group-Out cross-validation, random stratified subsets comprising 10 active compounds and 10 inactive compounds were removed from the training set in each cross-validation round, and the model was regenerated using the remaining compounds as training examples. The resulting model was used to predict the class label for the 20 removed compounds. The procedure was repeated 10 times, with each of the training set compounds removed at least once. The results were informed as the average percentage of good classifications (accuracy) across the folds, and this was compared to the accuracy of the model for the original training set and also, as advised by Gramatica (2013), to the No-Model error rate or risk (NOMER%), i.e., the error provided in absence of model:

$$\begin{array}{l} NOMER\%\;=\frac{\left(n\;-\;nm\right)}{n}\times 100 \end{array}$$

where *n* is the total number of objects and *nm* is the number of objects of the most represented class.

Finally, the predictivity of each individual model was assessed through external validation, using the 333-compound test set that was already described in section Dataset Collection. A diversity of statistical parameters commonly used to assess the performance of classificatory models (Roy and Mitra, 2011; Gramatica, 2013) were estimated for both the training and test sets: sensitivity (Se, i.e., true positive rate), specificity (Sp, i.e., true negative rate), accuracy (Acc, i.e., overall percentage of good classifications), positive and negative predictivity and the F-measure, which is defined as follows (Roy and Mitra, 2011):

$$\begin{array}{l} F-measure=\frac{2\times Se\times \left(1-Sp\right)}{Se\;+\;\left(1-Sp\right)} \end{array}$$

### Ensemble Learning

Classifier ensembles are known to provide better generalization and accuracy than single model classifiers (El Habib Daho and Chikh, 2015; Carbonneau et al., 2016; Min, 2016). Here, we have used two retrospective virtual screening campaigns to assess the performance of individual classifiers and classifier ensembles. As described in the next subsection, the first retrospective virtual screen allowed assessing the performance of individual classifiers and provided the basis to decide which individual models would be selectively combined in the model ensemble and how they would be combined. The second retrospective virtual screen served to the sole purpose of assessing the performance of the chosen model ensemble.

The best individual classifiers were selected and combined using the area under the ROC curve metric (AUC ROC) in the first retrospective screen as criterion of performance. To choose the ideal number of models to be included in the ensemble, systematic combinations of the 2 to 100 best performing classifiers were analyzed (the two best-performing models were combined, then the three best-performing models, the four best-performing models, and so on up to a total of 100 models included in the ensemble). Four combination schemes were applied to obtain a combined score: MIN operator (which returns the minimum score among the individual scores of the combined models); Average Score; Average Ranking and; Average Voting. Voting was computed according to the equation previously used by Zhang and Muegge (2006). AUC ROCs were obtained with the pROC package (Robin et al., 2011); the Delong method was used to statistically compare the AUC ROCs. BEDROC and RIE (1%) were also computed (Truchon and Bayly, 2007). For that purpose, we resorted to the R package enrichvs (enrichment assessment of virtual screening approaches; Yabuuchi et al., 2011) and the online tool ROCKER (Lätti et al., 2016).

### Retrospective Screening Campaigns

Through simulated ranking experiments, Truchon and Bayly (2007) demonstrated that the AUC ROC metric is dependent on the ratio of active compounds/inactive compounds, and the standard deviation of the metric converges to a constant value when small yields of actives (Ya) of the screened library are used (Ya below 0.05 seem to provide more robust results). Reasonably small Ya also ensures that the saturation effect is constant or absent. A high number of decoys (around 1,000 or higher) and a small Ya contribute to a controlled statistical behavior (Truchon and Bayly, 2007). Accordingly, to estimate the enrichment performance of our models and model ensembles in a real virtual screening scenario, we have performed retrospective virtual screening experiments. For that purpose, we have seeded known active compounds among a large number of decoys obtained with the help of the Directory of Useful Decoys Enhanced (DUD-E; Mysinger et al., 2012), a widely used benchmarking tool which allows the obtention of putative inactive compounds paired to known active compounds by physicochemical properties (e.g., molecular weight, logP, number of rotatable bonds, among others), but topologically dissimilar to such active compounds. In this way, two chemical libraries for such pilot screens were obtained. The first one, that we will call DUDE-A, was compiled by using the active compounds from the test set as queries in the DUD-E website. Such active compounds were later dispersed among the so-obtained paired decoys (putative inactive compounds). As a result, DUDE-A contained 31 known active compounds dispersed among 1500 DUD-E decoys and displayed a Ya of 0.020. DUDE-A was used to estimate the performance of the individual models in a virtual screening experiment and to choose the best individual models that would be included in the ensemble (i.e., to train the ensembles). It was also applied to choose which score threshold would be applied in prospective virtual screening campaigns. A second library, called DUDE-B, was obtained to validate the ensemble that showed the best performance in the DUDE-A screen. For that purpose, we compiled from literature 33 recently reported active compounds against FP-2 (IC50 ≤ 5 μM; Nizi et al., 2018; Stoye et al., 2019). The DUDE-B library was generated by merging these 33 active compounds with 4,337 decoys from the DUD-E website. The calculated Ya for DUDE-B is about 0.007.

### Building Positivity Predictive Value Surfaces and Choosing an Adequate Score Threshold Value

A practical concern when implementing *in silico* screens involves estimating the actual probability that a predicted hit will confirm its activity when submitted to experimental testing (Positive Predictive Value, PPV). Estimation of such probability is however precluded due to its dependency on the Ya of the screened library, which is not known *a priori*:

$$\begin{array}{l} PPV=\frac{Se\;Ya\;}{Se\;Ya+\left(1-Sp\right)\left(1-Ya\right)} \end{array}$$

where *Se* represents the sensitivity associated to a given score cutoff value and *Sp* represents the specificity. The former equation was applied to build PPV surfaces. In order to choose an optimal cutoff value to select predicted hits in prospective virtual screening experiments, 3D plots showing the interplay between PPV, the Se/Sp ratio and Ya were built for each individual model and for each model ensemble. This approach has recently been reported by our group (Alberca et al., 2018). Using DUDE-A (described in previous subsection), Se and Sp were computed in all the range of possible cutoff score values. Though there is no guarantee that the Se and Sp associated to each score value for DUDE-A will be the same when applying the classifiers to other libraries, e.g., in the prospective virtual screening campaign, since controlled statistical behavior is observed for database sizes of 1,000 compounds or more and Ya below 0.05, we can reasonably assume that the ROC curve and derived metrics will be similar when applying the models to classify other large chemical databases with low Ya. Taking into consideration that in real virtual screening applications Ya is ignored a priori but invariably low, Ya was varied between 0.001 and 0.010. The R package plotly (https://cran.r-project.org/package=plotly) was used to obtain all the PPV graphs. Visual analysis of the resulting PPV surfaces allowed us to select a score threshold value with a desired range of PPV.

### Prospective Virtual Screening

Based on visual inspection of the resulting of PPV graphs, we have applied in a prospective virtual screen an 11-model ensemble using the MIN operator to combine individual classifiers. Based on PPV surface analysis, we chose a score threshold that provides a PPV ≥ 20% at Ya = 0.01.

We have used the 11-model ensemble to screen two databases: (a) DrugBank 4.0, an online database containing extensive information about the US Food and Drug Administration (FDA) approved, experimental, illicit and investigational drugs (Law et al., 2014); (b) SWEETLEAD, a curated database of drugs approved by other international regulatory agencies, plus compounds isolated from traditional medicinal herbs and regulated chemicals (Novick et al., 2013). Both databases were curated using Standardizer 16.10.10.0 (ChemAxon). The following actions were applied to obtain homogeneous representations of the molecular structure for the subsequent virtual screen: (1) Strip salts; (2) Remove Solvents; (3) Clear Stereo; (4) Remove Absolute Stereo; (5) Aromatize; (6) Neutralize; (7) Add Explicit Hydrogens; and (8) Clean 2D. Duplicated structures were removed using Instant JCHEM v. 17.2.6.0. Four hits were selected for experimental evaluation, using the following criteria: (a) no previous report of falcipain-2 inhibition; (b) availability through local suppliers; (c) cost. Methacycline, benzthiazide, and bendroflumethiazide were acquired from Sigma-Aldrich. Odanacatib (99% HPLC) was acquired from AK Scientific (Y0388).

### Molecular Docking

To gain insight into the possible mode of action of the active hits, we studied their possible interactions with falcipain-2 by docking simulations. The structure of the enzyme was obtained from the Protein data bank. We retrieved the available experimental structure of the target in complex with an inhibitor, the epoxysuccinate E64, that shows 2.9 of resolution (PDB code: 3BPF; Kerr et al., 2009). Among the four chains crystalized, we selected the B chain for the simulations. We used AutoDockTools4 software to remove the inhibitor and the crystallographic water molecules off the pdb file, and to add the hydrogen atoms of the protein.

Autodock4 was used for docking simulations. The docking software and conditions were selected based on the previous investigation by Mugumbate et al. (2013). The authors showed the capacity of this software to replicate the experimental pose of E64 in the re-docking experiment and to identify known inhibitors from non-inhibitors through the docking scores. Our own results of the re-docking E64 into the binding site of FP-2 were similar to such previous investigations, since Autodock4 was able to reproduce the experimental pose with a RMSD value of 1.84 Å.

Odanacatib was docked into the active site of the enzyme. Calculations were conducted with a grid of 40X40X40 grid points, centered on the experimental ligand E64 (coordinates: −36.75, 31.05, −47.07 in x, y, and z, respectively) and with a spacing between grid point of 0.39. We used the default Autodock4 parameters for a population of 150, 50 genetic algorithm runs, 2.5 × 10^6^ evaluations and 27,000 maximum generations.

Regarding non-competitive inhibitor methacycline, we first used DoGSiteScore server (Volkamer et al., 2012) to detect possible binding pockets in the protein. The server proposed two regions of binding, besides the known catalytic binding site of the enzyme. Methacycline was docked in both regions and the best results were achieved in the area delimited by CYS39, SER41, TRP43, GLU67, GLN68, LEU70, VAL71, ASP72, CYS73, SER74, PHE75, ASN77, TYR78, GLY79, CYS80, TYR106, VAL107, SER108, ASP109, ALA110, PRO111, ASN112. The simulation was conducted in the conditions described before for odanacatib and E64, except for the position of the grid, which was centered on the side chain on VAL71, specifically in the carbon atom defined as CG1.

### Falcipain-2 Expression and Refolding

Falcipain-2 (MEROPS ID: C01.046) was expressed as inclusion bodies in BL21(DE3) *Escherichia coli* strain, purified by IMAC under denaturing conditions (final purity: 91%) and refolded to active enzyme as previously described (Pradines et al., 2001).

### Falcipain-2 Kinetic Assay

Falcipain-2 activity was assayed fluorometrically with Z-LR↓AMC (Bachem) as substrate in 100 mM acetate buffer pH 5.5 containing 5 mM DTT and 0.01% Triton X-100, as this is expected to increase enzyme stability and reduce the number of false positives (Jadhav et al., 2010). Assays (final reaction volume ~80 μL) were performed at 30°C in solid black 384-well plates (Corning) at fixed enzyme concentration (3.3 nM). Except stated otherwise, fluorogenic substrate was added at final concentration of 5 μM (~1 x K_M_) to match balanced assay conditions (Copeland, 2005). The release of 7-amino-4-methylcoumarin was monitored continuously for 60 min with a FilterMax F5 Multimode Microplate Reader (Molecular Devices) using standard 360 nm excitation and 465 nm emission filter set. Enzyme activity was estimated as the slope of the linear region of the resultant progress curves. Under the described conditions, falcipain-2 activity showed no significant changes in the presence of DMSO (0–8%) and the Selwyn test (Selwyn, 1965) indicated that enzyme remained stable during the assay.

### Falcipain-2 Inhibition Assay

1 μL of each compound (2.5 mM in DMSO), N-(trans-epoxysuccinyl)-l-leucine 4-guanidinobutylamide (E-64, Sigma-Aldrich) (10 μM in DMSO) or DMSO were dispensed into each well. Then, 40 μL of activity buffer containing falcipain-2 (6.6 nM) were added to each well, plates were homogenized (30 seg, orbital, medium intensity) and each well subjected to a single autofluorescence read (exc/ems = 360/465 nm). Plates were incubated in darkness for 15 min at 30°C and then 40 μL of Z-LR-AMC (10 μM in assay buffer) were added to each well to start the reaction. After homogenization (30 seg, orbital, medium intensity), the fluorescence of AMC (exc/ems = 360/465 nm) was acquired kinetically for each well (12 read cycles, one cycle every 300 s). Fluorescence measurements were used to determine the slope (dF/dt) of progress curves by linear regression and inhibition percentage (%Inh) was calculated for each compound according to:

$$\begin{array}{l} \%Inh=100\cdot \left[1-\left(dF/dt^{WELL}-\mu^{C-}\right)/\left(\mu^{C+}-\mu^{C-}\right)\right] \end{array}$$

where dF/dt^WELL^ represents the slope of each compound well and μ^C+^ and μ^C−^ the average of falcipain-2 + DMSO (no-inhibition) and substrate (no-enzyme) controls, respectively.

Compounds were re-tested in a dose-response manner (final concentration ranging from 375 μM to 44.7 pM) using identical assay conditions. 6 μL of compounds stock (10 mM in DMSO), E-64 (10 μM in DMSO), and DMSO were added to the first wells (column 1), followed by addition of 34 μL of activity buffer. After addition of 20 μL of buffer to subsequent wells, 24 serial 2-fold dilutions were made horizontally. Then, 40 μL of activity buffer containing falcipain-2 (6.6 nM) were added to each well, except for those corresponding to C-; completed with 40 μL of activity buffer. After homogenization, incubation, and autofluorescence measurement, 20 μL of Z-LR-AMC substrate (20 μM in activity buffer) were added. Data collection and processing were performed exactly as described above. At the concentration tested, no significant autofluorescence (360/465 nm) was apparent for the investigated compounds.

Percentage of falcipain-2 residual activity was calculated for each condition according to:

$$\begin{array}{l} \%Res.Act\;=100\cdot \left[\frac{\left(dF/dt^{WELL}-\mu^{C-}\right)\;}{\left(\mu^{C+}-\mu^{C-}\right)}\;\right] \end{array}$$

Half-maximal inhibitory concentration (IC_50_) and Hill slope parameters were estimated by fitting experimental data from dose-response curves to the four-parameter Hill equation by using GraphPad Prism program (version 5.03).

### Determining Reversibility, Mode of Inhibition, and Ki

Reversibility and time dependence of falcipain-2 inhibition by investigational compounds was assayed as previously described (Morrison, 1969). In brief, odanacatib (15 μM) and falcipain-2 (330 nM) were incubated at 30 °C for 60 min in activity buffer. Two microliters of the mix were rapidly added to 200 μL of Z-LR-AMC (5 μM in activity buffer) pre-incubated at the same temperature. Immediately after mixing, AMC fluorescence (λexc/ems = 355/460 nm, sensitivity = 550 V) was continuously monitored every second using a thermostated (30°C) Aminco Bowman Series 2 spectrofluorometer (Thermo Spectronic). In the case of the methacycline, the final inhibitor concentration in the mixture with falcipain-2 was 330 μM. For falcipain-2 control, the equivalent volume of DMSO vehicle was pre-incubated with the enzyme. To determine the kinetics of inhibition onset, falcipain-2 (3.3 nM final concentration) was added to 200 μL of reaction mix (previously tempered at 30°C) containing activity buffer, odanacatib (0.15 μM) and Z-LR-AMC (5 μM). Immediately after mixing, AMC release was monitored as indicated above. For methacycline (33 μM final concentration in the reaction mix), the experiment was exactly the same.

The identification of the mode of inhibition was performed as indicated previously. For odanacatib, falcipain-2 activity was determined for at least six different substrate concentrations (ranging from 62.5 to 1.95 μM) in the absence and presence of three fixed doses of inhibitor: 0.15, 0.5, and 2.5 μM. Data were re-arranged to estimate percentage of falcipain-2 residual activity for each condition and the values for IC_50_ and Hill slope were estimated by fitting experimental data to the four-parameter Hill equation by using GraphPad Prism. To estimate Ki, kinetic data were arranged in the form of Michaelis curves (dF/dt vs. [Z-LR-AMC]_0_) and globally fitted to the competitive inhibition equation present in GraphPad Prism (version 5.03). Finally, to estimate Ki by using the tight-binding inhibition approach (Morrison, 1969), data were transformed to fractional velocity vs. inhibitor concentration and re-analyzed by global fitting to the Morrison equation by using GraphPad Prism (version 5.03).

To identify the mode of inhibition of methacycline, dose-response curves (0–625 μM) were performed as described above at six different substrate concentrations (ranging from 1 to 50 μM) and fitted as indicated above to estimate the values of IC_50_ and Hill slope. Finally, kinetic data were arranged in the form of Michaelis curves (dF/dt vs. [Z-LR-AMC]_0_) and globally fitted to the mixed inhibition equation present in GraphPad Prism (version 5.03) for the simultaneous estimation of α and Ki.

### Determining the Sensitivity of Methacycline Inhibition to RedOx Potential

The inhibitory activity of decreasing concentrations (375 μM−91.6 pM) of methacycline were determined in activity buffer containing DTT (0.1–10 mM) or L-cysteine (0.1–10 mM) as indicated above. Resultant dose-response curves were fitted as previously indicated to estimate the values of IC_50_ and Hill slope.

### Densitometric Estimation of the Inhibition of Falcipain-2 Hemoglobinase Activity

Increasing concentrations of methacycline (200 μM, 500 μM, and 1 mM) and odanacatib (0.5, 5, and 50 μM) were preincubated with falcipain-2 (132 nM) for 30 min at 37°C in buffer 100 mM NaAc, 10 mM DTT pH 5.5. Then, human hemoglobin (H7379, Sigma-Aldrich) was added to a final concentration of 100 μg/mL to initiate reaction (final assay volume = 50 μL). E64 (10 μM) and DMSO were used as negative and positive controls, respectively. Also, a blank (no falcipain-2) control was included. In all cases, the final concentration of DMSO was 10%. Mixes were incubated without agitation for 3 h at 37°C to allow the enzymatic reaction to proceed. Then, reactions were stopped by addition of 15 μL of 5xSDS–PAGE sample buffer + 7.5 μL of DTT (1 M) and boiled for 5 min. Samples (22.5 μL, equivalent to 1.5 μg of hHb) were electrophoretically resolved by SDS-PAGE on a 15% acrylamide gel and Coomassie stained. The amount of undegraded hHb, observed as a doublet of around 15 kDa, was estimated densitometrically by using ImageJ 1.38d software (Nation al Institutes of Health, USA).

### Evaluation of Antiparasitic Activity

Human erythrocytes were obtained from volunteer donors with a procedure approved by CEIC (Committee for Ethics on Clinical Investigation, Facultad de Farmacia y Bioquímica, Universidad de Buenos Aires EXP-UBA: 0048676/2017). Human erythrocytes infected with the NF54 strain of *P. falciparum* were cultivated in RPMI 1640 medium supplemented with 0.5% albumax II (Invitrogen), 22 mM glucose, 25 mM HEPES, 0.65 mM hypoxanthine, and 50 mg/mL gentamicin. Cultures were maintained at 37°C by routine passage at 5% hematocrit with a maximum parasitemia of 5% in a 90% N_2_/5% O_2_/5% CO_2_ atmosphere as previously described (Alvarez et al., 2014).

When needed, ring-stage parasites were synchronized by using sorbitol treatment (Aley et al., 1986). After 24 h synchronization, cultures of infected erythrocytes at trofozoite-stage were treated with various concentrations of odanacatib, methacycline and E-64 for 48 h. Briefly, 100 μL of synchronous trophozoite-stage infected erythrocytes cultures were plated in 96-well at 4% hematocrit and 1% parasitemia. 100 μL of odanacatib (200, 20, or 2 μM), methacycline (1,000, 100, or 20 μM), E-64 (50, 10, or 2 μM), DMSO (vehicle control), or RPMI 1640 medium (control) were dispensed into each well to achieve final hematocrit of 2%, 0.5% parasitemia and the final concentration of each compound tested in a final volume of 200 μL. Parasitemia was evaluated by light microscopy counting infected forms of the parasite (ring, trophozoite, and schizont-stages) in a thick blood smear stained with Giemsa. A total of ~1,500 erythrocytes distributed in at least 15 random microscopic fields were evaluated of each smear and the parasitemia was calculated as (infected erythrocytes / total erythrocytes)^*^100. Then, each treatment was normalized to control parasitemia and expressed as percentage.

Significance was determined using one-way Analysis of Variance followed by a Tukey Multiple Comparison Test. Computations were carried out using PRISM statistical software (GraphPad Software, Inc., version 6). A *p*-value <0.05 was considered significant. The number of determinations (n) for independent preparations (N) are indicated.

## Results

A ligand-based virtual screening approach was used to discover falcipain-2 inhibitors. 1,000 individual linear classifiers were obtained by applying a random subspace approximation on a pool of more than 3,000 Dragon molecular descriptors. The individual models were internally and externally validated.

Results of the internal validation are shown in Table 1. Regarding the Leave-Group-Out, results for each individual classifier are informed as the average accuracy across the folds, which is in all cases above 80% and close to the correspondent accuracy on the training set, suggesting the models are robust. Since the proportions of the active and inactive compounds in the training set are identical (as are in each of the Leave-Group-Out folds) the correspondent NOMER% (associated to random classification) is 50%, well below the behavior of the models in the cross-validation.

**Table 1:** Results of the internal validation procedures for the best 11 individual classifiers.

**Model**	**Training set**	**LGO**	**Randomization**	
	**Acc (%)**	**Average Acc (%)**	**Confidence interval 95%**	
594	84.615	83.740	53.882	61.283
516	88.462	86.712	56.623	63.267
477	84.615	83.835	56.158	64.501
975	87.363	86.374	53.309	62.844
244	90.110	88.712	55.312	62.160
504	85.714	83.765	55.978	61.824
870	86.264	82.753	56.962	65.126
154	86.813	85.168	54.677	62.246
764	84.615	83.080	53.273	62.112
564	85.714	82.636	54.814	62.000
80	86.264	85.378	56.980	61.921

Regarding the randomization results, Table 1 shows the 95% confidence interval around the mean accuracy of the randomized models. As expected, the accuracy of the randomized models is in all cases much below the accuracy of the true (non-randomized) models, and very close to the NOMER%, suggesting a low probability of chance correlations for the true models.

External validation was performed using the 333-compound independent test set. The results are summarized in Table 2. In general, the individual classifiers show an acceptable performance. Due to the unbalanced nature of the test set (31 active compounds and 302 inactive ones) in comparison to the test set (91 active and 91 inactive compounds) some of the differences in the statistical parameters of the training and test sets are to be expected (i.e., decreased sensitivity in the test set, sharp drop in the positive predictivity and concomitant increase in the negative predictivity).

**Table 2:** Statistical parameters of the best individual classifiers, for both the training and test sets.

	**Training set**					
**Model**	**Se**	**Sp**	**Acc**	**F-measure**	**Positive predictivity**	**Negative predictivity**
Model 594	0.88	0.81	0.85	0.31	0.82	0.87
Model 516	0.89	0.88	0.88	0.21	0.88	0.89
Model 477	0.86	0.84	0.85	0.28	0.84	0.85
Model 975	0.89	0.86	0.87	0.25	0.86	0.89
Model 244	0.92	0.88	0.90	0.21	0.88	0.92
Model 504	0.87	0.85	0.86	0.26	0.85	0.87
Model 870	0.89	0.84	0.86	0.28	0.84	0.88
Model 154	0.88	0.86	0.87	0.25	0.86	0.88
Model 764	0.84	0.86	0.85	0.24	0.85	0.84
Model 564	0.85	0.87	0.86	0.23	0.87	0.85
Model 80	0.89	0.84	0.86	0.28	0.84	0.88
	**Test set**					
Model 594	0.84	0.80	0.80	0.32	0.30	0.98
Model 516	0.77	0.82	0.82	0.29	0.31	0.97
Model 477	0.71	0.84	0.83	0.26	0.31	0.97
Model 975	0.87	0.80	0.81	0.32	0.31	0.98
Model 244	0.84	0.77	0.78	0.36	0.28	0.98
Model 504	0.81	0.81	0.81	0.30	0.31	0.98
Model 870	0.81	0.81	0.81	0.31	0.30	0.98
Model 154	0.71	0.82	0.81	0.29	0.29	0.96
Model 764	0.74	0.83	0.83	0.27	0.32	0.97
Model 564	0.77	0.84	0.83	0.27	0.33	0.97
Model 80	0.81	0.75	0.76	0.38	0.25	0.97

*The default score cutoff value (0.5) was used to discriminate between active and inactive compounds and estimate the parameters. Note that this score has been later optimized to obtain improved Se/Sp relationships*.

The best individual model included the following features:

Model 594

Class = −0.48333 + 0.38415^*^SM08_AEA(bo) - 6.50601^*^SpPosA_A - 0.12786^*^C-005 + 0.35800^*^B05[N-N] + 0.13459^*^nR = Cs + 0.21576^*^CATS2D_02_DD + 0.25881^*^nS(=O)2 - 0.33510^*^B03[O-S] - 0.07816^*^N-072 + 0.16832^*^B06[C-S]

Wilks' Lambda:.45705 approx. *F*_(10, 171)_ = 20.314 *p* < 0.0000. Dragon's nomenclature for the molecular descriptors has been kept in the previous expression. SM08_AEA(bo) corresponds to the spectral moment of order 8 from augmented edge adjacency matrix weighted by bond order; SpPosA_A is the normalized spectral positive sum from adjacency matrix; C-005 refers to the frequency of CH3X groups where X indicates an electronegative atom (O, N, S, P, Se, halogens); B05[N-N] indicates the presence/absence of the N – N pair at topological distance 5; nR = Cs refers to the number of aliphatic secondary C(sp2); CATS2D_02_DD is the CATS2D Donor-Donor at lag 02; nS(=O)2 symbolizes the number of sulfones; B03[O-S] indicates the presence/absence of O – S pair at topological distance 3; N-072 refers to frequency of the atom-centered fragment RCO-N < / >N-X = X; B06[C-S] denotes the presence/absence of a C – S pair at topological distance 6. The molecular descriptors associated to presence/absence or frequency of a given feature indicate differences in the frequencies at which such features appear in the active and inactive class of the training set. Those descriptors associated in the model to a positive weighting coefficient (B05[N-N], nR = Cs, nS(=O)2, and B06[C-S]) show that such feature is more frequent in the active compounds than in the inactive ones. In contrast, the descriptors associated to a negative coefficient (SpPosA_A, C-005, and N-072) indicate that such features appear more frequently in the compounds of the inactive class than in the ones of the active class. CATS2D_02_DD is a two-dimensional Chemically Advanced Template Search descriptor similar to a pharmacophore pair (Reutlinger et al., 2013), but considering topological distances between the pharmacophore points instead of geometrical distances. Here, the descriptor suggests that two H-bond donors at a topological distance of two are a desirable feature in falcipain-2 inhibitors.

The physicochemical interpretation of the two descriptors associated to spectral moments of a topological matrix (SM08_AEA(bo) and SpPosA_A) is less immediate.

An augmented edge adjacency matrix aE(w) is a symmetric square matrix that can be derived from an edge-weighted molecular graph, for any weighting scheme w. The elements from such matrix [aEw]ij take values of 1 if i and j are adjacent edges/bonds, values wi if i equals j (that is, for the elements in the diagonal) and values of 0 otherwise (i.e., for non-diagonal elements corresponding to non-adjacent edges; Liu et al., 2018). The *k*th spectral order μ_k_ of a topological matrix M can be defined as:

$$\begin{array}{l} \mu_{\text{k}}\;=\text{ tr}\left({\text{M}}^{\text{k}}\right) \end{array}$$

where k is the power of the matrix and tr is its trace, i.e., the sum of the diagonal elements (Estrada, 1996). The kth spectral moment of the edge adjacency matrix has a simple graph theoretical interpretation (Estrada, 1996): it is the sum of all self-returning walks of length k in the line graph of the molecular graph, beginning and ending with the same vertex. It may then be appreciated that the value of such descriptor would be highly influenced by the presence of ring systems and the nature of such cycles (e.g., fused rings). Since the considered augmented edge matrix is weighted by the bond order, the presence of double and triple bonds and aromatic systems will tend to increase the value of the descriptor. Generally speaking, active examples in our training set tend to display higher values of SM08_AEA(bo).

Regarding SpPosA_A, it denotes the normalized sum of positive eigenvalues of the adjacency matrix. Its value diminishes with increase branching, with greater emphasis in terminal rather than in central branching (Balaban et al., 1991).

The 11 best individual models and a brief description of the descriptors included in them have been listed as Supplementary Data Sheet 2.

For a more challenging and realistic simulation, the enrichment behavior of the individual models was studied through a retrospective virtual screen on DUDE-A library, where a small proportion of active compounds (31) was dispersed among a high number (1500) of putative decoys. Initially, we compute the area under the Receiver Operating Characteristic curve (AUC ROC) to assess the classificatory performance of the models. 100, 93.4 and 3.1% of the individual classifiers displayed AUC ROCs above 0.8 for the training set, the test set and the DUD-A library, in that order. 85.8% of the individual models achieved an AUC ROC > 0.90 for the training set, whereas only one of the models (named model 975) got an AUC ROC above 0.9 for the test set, none of the models achieved an AUC ROC above 0.9 for the DUDE-A library. This suggests that our random subspace approach has been successful in finding individual classifiers with good explanatory and predictive power, but also that some degree of overfitting may also be present. Results also suggests that the retrospective screening experiment on DUDE-A is the more challenging tasks for the classifiers. Table 3 shows the 11 individual classifiers that showed the best performance on the DUDE-A library, along with their AUC ROC, BEDROC, and RIE values.

**Table 3:** Values of the AUC ROC, BEDROC, and RIE metrics for the 11 individual classifiers that displayed the best performance on the DUDE-A library.

**Model**	**AUC ROC**			**BEDROC (α = 20)**	**RIE 1%**
	**Training set**	**Test set**	**DUDE-A**	**DUDE-A**	**DUDE-A**
594	0.9162	0.8819	0.8529	0.2565	0.0000
516	0.9396	0.8819	0.8508	0.3150	0.0000
477	0.9123	0.8910	0.8419	0.2647	3.2258
975	0.9475	0.9105	0.8355	0.2191	3.2258
244	0.9492	0.8599	0.8343	0.2597	3.2258
504	0.9343	0.8812	0.8314	0.2355	0.0000
870	0.9291	0.8776	0.8294	0.2195	0.0000
154	0.9275	0.8730	0.8283	0.2769	3.2258
764	0.9195	0.8810	0.8268	0.2361	0.0000
564	0.9271	0.8992	0.8205	0.1673	0.0000
80	0.9233	0.8478	0.8198	0.1969	0.0000

**Figure 2:**
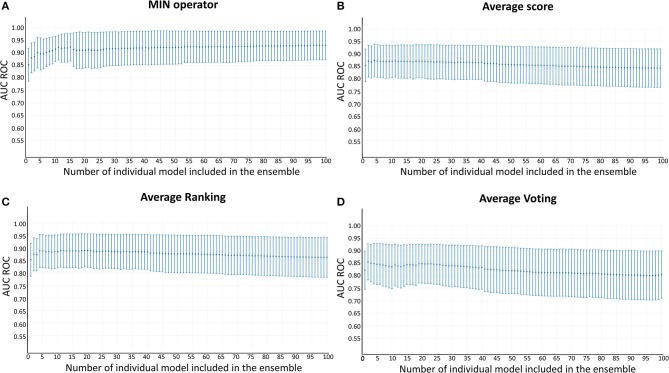
AUC ROC metric vs. the number of combined models in the DUD-E database. **(A)** Minimum score; **(B)** Average score; **(C)** Average ranking; **(D)** Average voting.

Whereas, the performance of the best individual classifiers was quite satisfactory, we explored ensemble learning approaches to obtain meta-classifiers with improved accuracy and a more robust behavior. Figure 2 shows the AUC ROC values (DUDE-A) obtained when systematically combining between the 2 and 100 individual models that displayed the best performance on the DUDE-A library, using four combination schemes: Minimum score (MIN), average score (AVE), average ranking (RANK), and average voting (VOT). The expectations on the ensembles were confirmed statistically: two combination schemes (minimum of the best 11 models and average ranking of the 4 best models) statistically outperformed the individual models in the DUDE-A database (*p* = 0.0003 and *p* = 0.0132, in that order). The MIN operator consistently outperformed the other combination schemes. When considering the influence of the number of models combined by the MIN operator on the AUC ROC metric, it was observed that above 11 models the AUC ROC did not improve substantially but poorer statistical behavior in terms of the standard deviation of the mean estimation was observed. The enrichment metrics for the best ensembles are shown in Table 4. Note that when applied in the screening of the DUDE-B database, the enrichment metrics for the 11-model combination based on the MIN operator (MIN-11) are similar (or in some cases, even better) than when screening DUDE-A database, validating the enrichment power of the best model combination. It may also be observed that best ensemble achieved good to excellent enrichment metrics. For instance, in the DUDE-A library, the RIE metric indicates that among the top 15 ranked compounds, 10 are known active ones.

**Table 4:** Values of the AUC ROC, BEDROC, and RIE metrics for the best model combination (DUDE-A library).

	**AUC ROC**		**BEDROC (α** **=** **20)**		**RIE (1%)**	
**Model**	**DUDE A**	**DUDE B**	**DUDE A**	**DUDE B**	**DUDE A**	**DUDE B**
MIN-11	0.9214^**^	0.8991^**^	0.6414	0.4252	29.6322	9.0289
M-594	0.8529	0.7415	0.2565	0.2710	0.0000	0.0000

*For comparison purposes, the results of the best individual model (M-594) are also presented, as well as the enrichment metrics for the MIN-11 ensemble on the DUDE-B library*.

** *Statistically significant differences in comparison with the best individual model (p < 0.01)*.

Based on the previous results, we chose to move to the prospective (real) virtual screening campaign with the combination scheme based on the MIN operator (MIN-11). In our experience, this model combination scheme leads to high-specific model combinations (i.e., small false positive rate), which is a particularly convenient approach in our context (a small academic group from a low- to mid-income country, with limited resources to invest in hit validation); we thus often prefer to reduce the false positive rate even if this means losing sensitivity and sacrificing some active scaffolds. We have chosen to refine the former criteria (prioritizing Sp) by resorting to PPV surface analysis (Alberca et al., 2018). With the help of PPV surfaces, the evolution of the most relevant metric for our purposes, the PPV, i.e., the actual probability that a predicted hit will confirm activity when submitted to experimental testing, can be visually optimized as a function of the (Se/Sp) ratio across a range of Ya values. For this analysis, we have considered that the association between the Se/Sp and the score values of the MIN-11 model ensemble (observed in the retrospective screening campaign on DUDE-A) will hold when performing screens on other libraries (e.g., in a prospective virtual screening application). This is a strong assumption that of course is not necessarily true. However, since the AUC ROC values obtained for the DUDE-A library are unmistakably high (above 0.9 for the best model ensemble) while on the other hand the DUDE-A database Ya ratio (0.02) and size (>1,000 compounds) speak of a controlled statistical behavior (Truchon and Bayly, 2007), we believe it is a reasonable assumption in the present setting.

**Figure 3:**
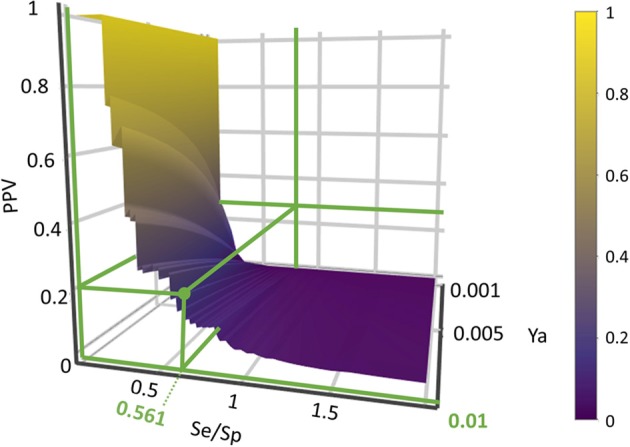
PPV surface for the best 11-model ensemble on the DUDE-A library. The Se/Sp ratio correspondent to the chosen score cut-off value and the associated PPVs within the Ya 0.001–0.010 range are signaled.

**Figure 4:**
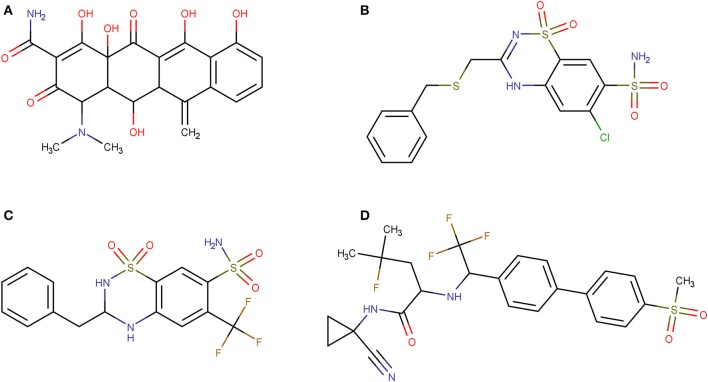
Molecular structures of the hits selected in the prospective virtual screening campaign that were submitted to experimental confirmation. **(A)** methacycline; **(B)** benzthiazide; **(C)** bendroflumethiazide; **(D)** odanacatib.

Using PPV surfaces (Figure 3), we chose 0.58 as score threshold to be used in our prospective virtual screening campaign; such score is associated to a Se/Sp ratio of 0.561 for MIN-11, and to a PPV value ≥ 20% for a Ya of 0.01. This means that if Ya in the real virtual screen was 0.01, we would have to submit about five predicted hits to experimental testing in order to find one confirmed hit. The virtual screen using the previous score cutoff value resulted in 157 hits, with 72 of them corresponding to approved drugs. Based on the previous analysis and our funding availability, we acquired and submitted four hits (Figure 4) to experimental testing: the antibiotic methacycline, the antihypertensives benzthiazide and bendroflumethiazide, and the abandoned drug odanacatib (an inhibitor of the cysteine protease cathepsin K that was pursued as a treatment for osteoporosis and bone metastasis but whose development was abandoned at Phase III long-term clinical trials due to safety issues; Drake et al., 2017).

To evaluate the ability of the selected hits to inhibit falcipain-2, we performed a two-round screening strategy. First, compounds were assayed in single dose (31.25 μM) to discard inactive molecules. Given that all of them were able to reduce to some extent (6–85%) the activity of falcipain-2 in comparison with the DMSO vehicle; we decided to evaluate the four hits in a dose-response manner (375 μM−0.45 pM) under balanced assay conditions to equalize the chances to detect competitive, non-competitive and uncompetitive inhibitors (Copeland, 2003; Yang et al., 2009). At the excitation/emission wavelengths used for AMC recording, compounds showed no significant autofluorescence in the concentration range tested. Prior to the analysis of the complete data, we explored the correlations between inhibition percentages in the primary (31.25 μM) and secondary (23.4 μM) screenings.

**Figure 5:**
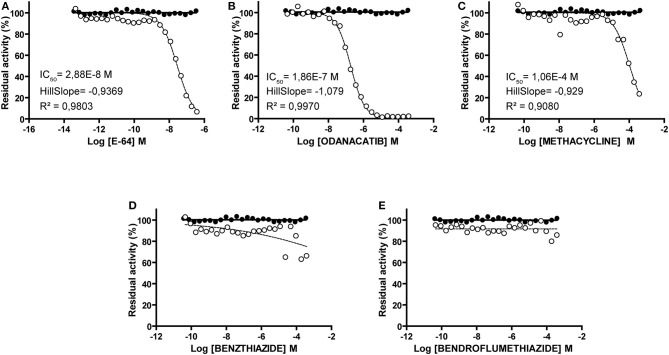
Dose-response curves of identified falcipain-2 inhibitors (open circles). For each compound, dotted line represents the best fit of experimental data to the four-parameter Hill equation. **(A)** E-64. **(B)** Odanacatib. **(C)** Methacycline. **(D)** Benzthiazide. **(E)** Bendroflumethiazide. For those compounds achieving data convergence, the resultant values for the parameters IC50, Hillslope and R2 are indicated. In all cases, equivalent volumes of DMSO vehicle were assayed in parallel (closed circles).

Compounds showed consistent results in both screenings (correlation coefficient *r*^2^ = 0.98; slope = 0.9732; Supplementary Data Sheet 3), with odanacatib and methacycline being the most active. These compounds showed typical progression (Supplementary Data Sheet 3) and dose-response curves (Figure 5), with measurable IC_50_ and Hill slope values of 0.186 μM and −1.079 for odanacatib, and 106.4 μM and −0.9294 for methacycline. In the same range of concentrations, benzthiazide and bendroflumethiazide showed no dose-dependent inhibition.

**Figure 6:**
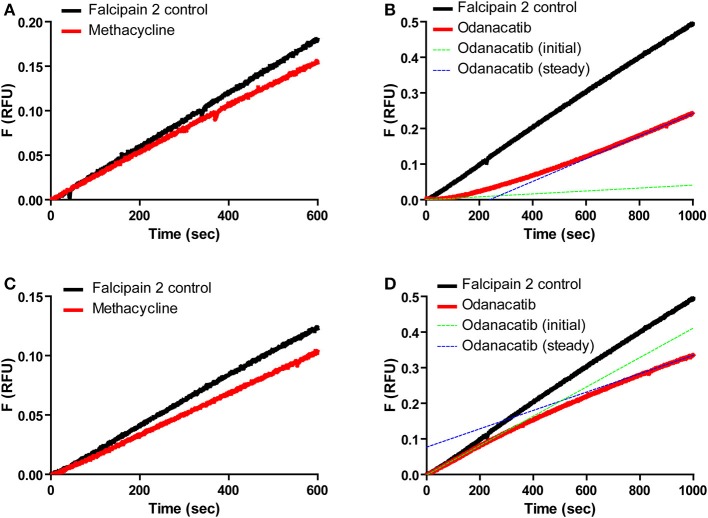
Reversibility and time dependence of the inhibition of falcipain-2 by methacycline and odanacatib. Top panel: Product progress curves for the dissociation of E-I complex by rapid dilution (100-fold) of enzyme-inhibitor mix into substrate solution. **(A)** Methacycline. **(B)** Odanacatib. Bottom Panel: Product progress curves for the formation of E-I complex by rapid addition of enzyme to a substrate-inhibitor mix. **(C)** Methacycline. **(D)** Odanacatib.

We further characterized odanacatib and methacycline in terms of the reversibility and time-dependence of falcipain-2 inhibition. Reversible interaction with falcipain-2 was verified for both compounds by the recovery of enzyme activity after rapid addition of substrate (100-fold dilution) to the pre-incubated mix of enzyme and inhibitor (Figures 6A,B). In this experiment, methacycline displayed a linear progress curve (Figure 6A) with a stable inhibition value, indicative of rapid onset of steady state (i.e., rapid dissociation of EI complex). In the presence of odanacatib, however, the enzyme took several minutes to recover full activity and to show a permanent inhibition value (concave progress curve), suggesting slow dissociation of inhibitor from the complex with falcipain-2 (Figure 6B). Similarly, both inhibitors displayed different kinetic behavior when enzyme was added to a reaction mix previously containing inhibitor and substrate (Figures 6C,D). Methacycline displayed a typical linear progress curve (Figure 6C), showing a defined (stable) value of inhibition during the whole assay. In contrast, odanacatib showed non-linear kinetics (Figure 6D) with inhibition progressively increasing over time (time-dependent inhibition). As stable inhibition was observed only after ~15 min, all subsequent kinetic experiments for odanacatib included preincubation (≥ 30 min at 30°C) with the enzyme.

**Figure 7:**
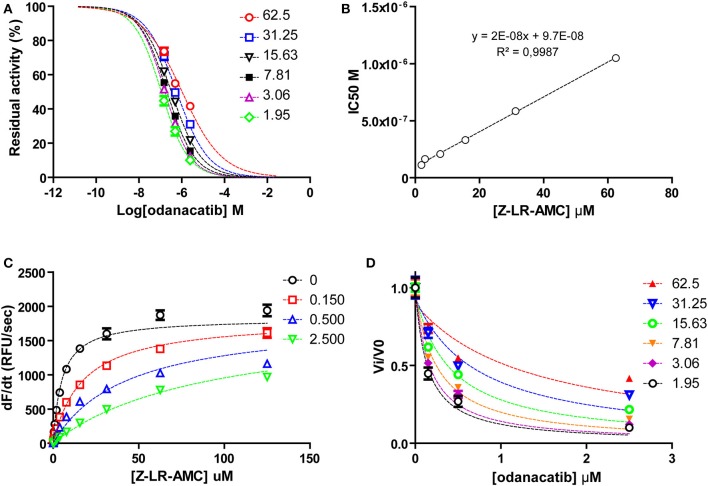
**(A)** Dose-response curves for odanacatib at fixed substrate concentrations. Dotted lines represent the best fit of experimental data to the four-parameter Hill equation. **(B)** Effect of substrate concentration on the IC50 values of falcipain-2 inhibition by odanacatib. IC50 values increase linearly (>9-fold) with substrate concentration in the range 1.95–62.5 μM. Dotted line represents the best fit of data to linear equation. Y-axis intercept accounts for the Ki value. **(C)** Global fitting of kinetic data to the competitive inhibition model equation. **(D)** Global fit ting of kinetic data to the Morrison equation.

To investigate the mode of inhibition of odanacatib, we first evaluated the impact of substrate concentration on the apparent IC_50_ value over a wide range (0.4–13.2xK_M_) of substrate saturation levels. For this, we used a reduced set of three odanacatib concentrations selected to: (i) include IC_50_ value at each substrate condition and (ii) cover the wider inhibition range (~15–80%) in the central stretch of the dose-response curves. As observed in Figures 7A,B, apparent IC_50_ values increased linearly with the increment of substrate concentration, indicating a competitive mode of inhibition for odanacatib on the activity of falcipain-2. The global fitting of all the Michaelis curves to the equation of competitive inhibition (Figure 7C) allowed us to estimate a Ki value of 98.2 ± 10.2 nM. As this estimation is in the limit of tight-binding inhibition (Ki ≤ 10^−7^ M), kinetic data were transformed to fractional velocity vs. inhibitor concentration and re-analyzed by global fitting to the Morrison equation^32^. Ki value for odanacatib determined from this approach (Figure 7D) was 99.88 ± 8.28 nM, very similar to our previous (more approximate) estimation.

**Figure 8:**
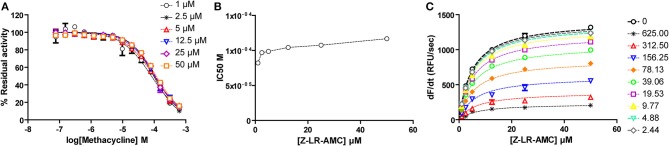
Methacycline is a non-competitive, sub-millimolar inhibitor of falcipain-2. **(A)** Dose-response curves for methacycline at fixed substrate concentrations. Dotted lines represent the best fit of experimental data to the four-parameter Hill equation. **(B)** Half maximal inhibitory concentration of methacycline increases slightly (1.42-fold) with substrate concentration in the range: 1–50 μM. **(C)** Global fitting of kinetic data to the equation of mixed inhibition model.

For methacycline, which initially showed potency in the high micromolar range, we constructed complete dose-response curves at six fixed substrate concentrations, ranging from 0.2 to 10xK_M_. Although substrate concentration was increased 50-fold, only a slight increase (1.42-fold) was observed in the apparent IC_50_ value, suggesting no competition between methacycline and the small peptidic substrate Z-LR-AMC. To directly estimate Ki and α values, Michaelis plots were globally fitted to the model for mixed inhibition. This approach confirmed that methacycline inhibits falcipain-2 activity non-competitively with a Ki value of 84.4 ± 6.5 μM and α = 1.42 ± 0.15 (Figure 8).

Given that cysteine peptidases require that its catalytic sulfhydryl group be in reduced state to show their maximal enzymatic activity, they are prone to undergo RedOx interferences caused by several classes of thiol-reactive compounds able to simulate genuine inhibition (Thorne et al., 2010). In many cases, this artifactual inhibition can be significantly relieved by simply changing the reduction potential of the activity buffer, thus providing a diagnostic test to detect false-positive RedOx compounds. To establish if this could be the case for methacycline, we further investigated the effect of the strength and concentration of reducing agents (DTT, a strong reducing agent, and cysteine, a weak reducing agent) on the inhibition of falcipain-2 by this molecule. Dose-response curves were very similar regardless of the final concentration of the reducing agent (100-fold range) present in the assay buffer (Supplementary Data Sheet 3). These results rule out common types of RedOx interference and suggest that methacycline genuinely inhibits falcipain-2.

Once established that odanacatib and methacycline inhibit the peptidolytic activity of falcipain-2, we assayed whether these molecules could also modulate falcipain-2 proteolytic activity on its natural substrate, human hemoglobin (hHb). To this end, we pre-incubated the enzyme with increasing concentrations of both inhibitors and then added the hHb substrate. E-64, a specific and highly potent irreversible inhibitor of C1A cysteine peptidases, was used as a positive control. After incubation at 37°C for 3 h, reaction mixes were resolved electrophoretically by SDS-PAGE on a 15% acrylamide gel and Coomassie stained. The amount of undegraded hHb, observed as a doublet of around 15 kDa, was estimated densitometrically. As shown in Supplementary Data Sheet 3, odanacatib inhibited the hemoglobinase activity of FP2 in a dose-response manner. For methacycline, however, no inhibition was observed in this assay, even at the highest concentration tested (1 mM). Of note, the effective inhibitory concentrations of odanacatib in this assay were in the low-to-middle micromolar range, a significant shift in comparison to the sub-micromolar potency previously observed in the inhibition of Z-LR-AMC hydrolysis. Overall, these observations suggest that (i) the existing differences between the surrogate (peptidic) and the natural (macromolecular) falcipain-2 substrates are somehow important for the inhibitory efficiency of both inhibitory molecules and that, at least, (ii) low-to-middle micromolar compound concentrations would be required to assess their efficacy in a more physiological context (i.e., cellular culture).

**Figure 9:**
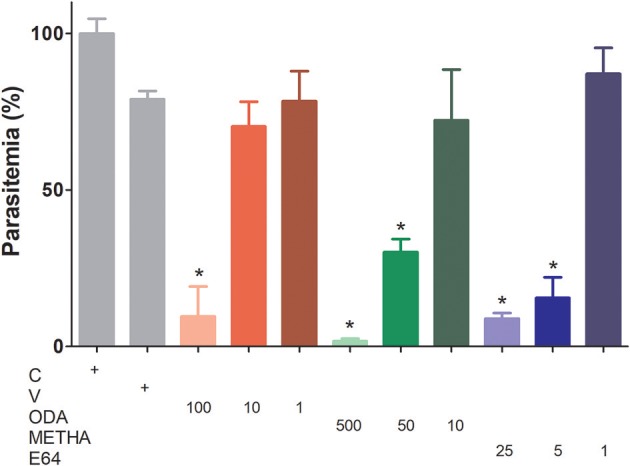
Effect of odanacatib and methacycline on the development of P. falciparum under culture. Cultures of erythrocytes infected (trophozoite stage) at 2% hematocrit and 0.5% parasitemia were incubated with increasing concentrations of odanacatib (ODA; 1, 10, or 100 μM) and methacycline (METHA; 10, 50, or 500 μM). E-64 (1, 5, or 25 μM) was used as positive control of falcipain-2 inhibition. DMSO was used as a vehicle control (V) and RPMI 1640 medium as a control (C). After 48 h, the number of infected erythrocytes (ring, trophozoite, and schizont-stages) was evaluated by light microscopy in stained blood smears. Data are the means ± SD of one experiment performed by triplicate. (^*^) *p* < 0.05 represents the differences between control and the treatments.

To analyze the influence of odanacatib and methacycline in the intraerythrocytic cycle of *P. falciparum*, a synchronized culture of RBCs infected (trophozoite stage) was treated with increasing concentrations of odanacatib (1, 10, or 100 μM) and methacycline (10, 50, or 500 μM). E-64 (1, 5, or 25 μM) was again used as a positive control. After 48 h, the number of infected erythrocytes was evaluated by light microscopy in stained blood smears. Odanacatib (100 μM) significantly reduced the parasitemia (Figure 9), with no apparent reduction in the other two concentrations tested. Methacycline significantly reduced the parasitemia at 500 and 50 μM, but not at 10 μM. As expected, E-64 significantly reduced the parasitemia at 5 and 25 μM, but no at 1 μM. Almost no erythrocytes infected at schizont-stage were observed in the treatments (Supplementary Data Sheet 3). It is important to mention that at the highest concentrations assayed, methacycline (500 μM) and odanacatib (100 μM) induced cytotoxic effects on RBC, as observed in the hemolysis assay (Supplementary Data Sheet 3).

**Figure 10:**
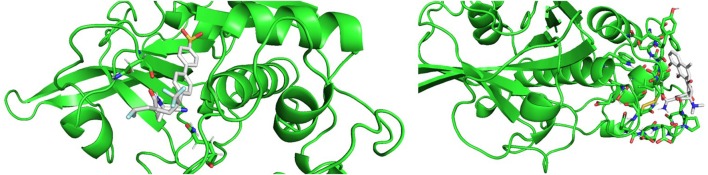
Best poses from the molecular docking experiments for odanacatib **(Left)** and methacycline **(Right)**. Interacting residues are shown in sticks.

Molecular docking results were in good agreement with the experimental observations. Figure 10 shows the best result of the docking simulation for odanacatib in the catalytic binding site, that is, the pose that showed lower value of the scoring function. Two hydrogen bonding interactions were found between the compound and GLN36 and ASN173. The docking score was −6.94 kcal/mol, which is lower than the score achieved for E64 in the same conditions (−4.91 kcal/mol). Regarding methacycline, we detected hydrogen bonding interactions between methacycline and the residues of the proposed (non-catalytic) binding pocket. Residues like ASP72, ASN112, PRO111, and ALA110 could be implicated in the stabilization of the complex (docking score of−5.65 kcal/mol). More studies will be performed to evaluate these predictions.

## Discussion and Conclusions

Using an ensemble learning approximation, we have performed a ligand-based virtual screening campaign to identify new falcipain-2 inhibitors as potential new treatments against malaria. There are some previous reports on the development of ligand-based models to predict the activity of *P. falciparum* cysteine proteases. The approaches used in such studies show considerable differences with the one reported here: they used conformation-dependent descriptors (3D QSAR) to infer regression models; in almost all cases, congeneric series of comparatively narrow chemical diversity have been used to train the models, thus limiting their applicability domain and; the reported models have mostly been used for explanatory rather than predictive purposes. Xue and coworkers realized CoMFA and CoMSIA 3D-QSAR studies on a series of 93 alkoxylated and hydroxylated chalcones (Xue et al., 2004). Potshangbam and coworkers also carried out CoMSIA and CoMFA studies on a series of 54 2-pyrimidinecarbonitrile analog inhibitors of falcipain-3 (Potshangbam et al., 2011). Using the same approximations, Wang et al. performed a 3D QSAR study of 247 2-pyrimidinecarbonitrile analog inhibitors of falcipain-3 (Wang et al., 2013). Teixeira and colleagues did a CoMFA and CoMSIA analysis of a series of 39 peptidyl vinyl sulfone derivatives as potential cysteine protease inhibitors (Teixeira et al., 2011). Very recently, Allangba et al. derived complexation QSAR models and pharmacophores from a training set of 15 lactone–chalcone and isatin–chalcone hybrid inhibitors with falcipain-2 inhibitory activity (Allangba et al., 2019). The most similar study to our own is possibly the one by Mugumbate and coworkers, who as a part of and hybrid ligand- and structure-based approach, obtained ligand-based models based on Pentacle alignment-independent descriptors, using a training set of nine non-peptide inhibitors of falcipain-2 (Mugumbate et al., 2013). They performed a retrospective pilot screen before using their protocol to explore the ZINC database, retrieving falcipain-2 inhibitors in the low μM range. All in all, the enrichment metrics they computed in their retrospective screen are similar to the ones we obtained here.

Our most predictive model combination was chosen (i.e., trained) based on a retrospective virtual screening campaign (DUDE-A library). The enhanced ability of the selected model-ensemble to retrieve falcipain-2 inhibitors in comparison to our best individual models was checked using a second retrospective virtual screening experiment (DUDE-B library). Four of the hits emerging from our prospective virtual screening experiment were acquired and assayed against the enzyme. Two of them, odanacatib (previously investigated as treatment against osteoporosis and bone metastasis) and methacycline (an antibiotic) confirmed our predictions, reducing the peptidolytic activity of the enzyme. Interestingly, our observed PPV (50%, corresponding to two experimentally confirmed hits out of four assayed compounds) exceeded our theoretic expectations based on PPV surfaces analysis, which suggested a PPV of at least 20% for the chosen score threshold, for a hypothetic yielding of active compounds of 1%.

Both hits displayed different inhibition mechanisms. In agreement with previous reports for the interaction with other Papain-like C1A (Clan CA family) human cathepsins (Gauthier et al., 2008), odanacatib inhibits falcipain-2 in a reversible, competitive and tight-binding (sub-micromomlar) mode. For human cathepsin K, odanacatib inhibition occurs throughout the formation of a covalent but yet reversible thioimidate adduct between the -SH in the catalytic Cys residue and the nitrile warhead (Oballa et al., 2007). This covalent association mechanism results in on- and off-rates of 5.3 ×10^6^ M and 0.0008 s^−1^ (t_1/2_ ~14 min), respectively (Gauthier et al., 2008). These observations are in line with the slow association and dissociation kinetics observed by us for falcipain-2 inhibition, suggesting that a similar chemical inhibition mechanism could be occurring. Other compounds bearing the N-(1-cyanocyclopropyl)-amide inhibitory scaffold present in odanacatib, have been reported as potent (Ki ~1–2 nM) and selective (>15-fold over human cathepsins) falcipain-2 inhibitors (Ang et al., 2011; Nizi et al., 2018).

On the other hand, methacycline acts as a reversible, non-competitive and sub-milimolar inhibitor of falcipain-2. Based on our observations (reproducible, reversible and dose-dependent reduction of enzyme activity, rapid equilibrium onset, Hill slopes ~ −1, inhibitory activity insensitive to the RedOx potential and no signs of common compound-specific assay interferences such as autofluorescence or aggregate formation), methacycline inhibition seems to occur throughout a genuine mechanism. This may lead to rational optimization efforts to improve affinity. Further studies should be performed to confirm the putative binding pocket suggested by our docking experiments, to move in that direction. To date, only few non-competitive falcipain-2 inhibitors have been reported; including suramin analogs (Marques et al., 2013), heme analogs (Marques et al., 2015), and (E)-chalcones (Bertoldo et al., 2015) reported. Suramin, heme, and their analogs inhibit falcipain-2 with IC_50_ values in the nanomolar range and seem to share a common “non-competitive like” inhibition mechanism that occurs through the formation of a ternary enzyme:inhibitor:substrate complex of stoichiometry 1:1:2. In both cases, the authors argued that the binding of the inhibitor to falcipain-2 reveals a novel regulatory substrate binding site in the enzyme, which allows the subsequent allosteric binding of a second substrate molecule, resulting in falcipain-2 inhibition (Marques et al., 2013, 2015). Very similar to what we have found for methacycline, (E)-chalcones 48 (Ki = 45 μM, α <1) and 66 (Ki = 7 μM, α = 1) display a classical non-competitive inhibition profile for falcipain-2 with no evidence of substrate inhibition (Bertoldo et al., 2015). None of these inhibitors appear to bind falcipain-2 active site, thus anticipating new routes to overcome the critical issue of selectivity over human cathepsins. In this regard, the identification and targeting of non-active (i.e., allosteric) binding sites within the falcipain-2 molecule seems to be an attractive and effective alternative to traditional active site-directed inhibitors, as recently showed by Pant and coworkers (Pant et al., 2018). Two compounds, rationally designed to target an allosteric site present in the pro-mature domain interface of falcipains-2/-3, were able to bind both pro-enzymes with nanomolar affinities and arrest *P. falciparum* growth, clearly illustrating the potential of this approach.

Of interest, odanacatib inhibits the peptidolytic activity of falcipain-2 much more efficiently than its hemoglobinase activity. Although displaying comparable affinities for the enzyme [${\text{K}}_{\text{D}}^{\text{hHb}}$ = 3.3 μM (Hogg et al., 2006); ${\text{K}}_{\text{M}}^{\text{Z}-\text{LR}-\text{AMC}}=$ 4.8 μM], there are important differences between Z-LR-AMC and hHb as falcipain-2 substrates, regarding both their binding modes and their catalytic heterogeneity. In the first case, the small peptidic substrate Z-LR-AMC accommodates completely within falcipain-2 active site and the enzyme-substrate complex relies entirely on active site interactions for its stabilization and catalytic transformation. In addition, Z-LR-AMC substrate comprise a single cleavage site per molecule. These facts seem to make it somehow more vulnerable to the competitive binding of odanacatib to the active site and to the catalytic impairment promoted by the binding of methacycline to a presumptive allosteric site, like the one proposed here. In the case of hHb, it has been shown that interaction with the enzyme depends almost exclusively on a unique falcipain-2 structural motif (the “arm”), located >25 Å away from the active site (Pandey et al., 2005; Wang et al., 2006). In fact, a falcipain-2 mutant lacking most of the arm loop showed no activity or affinity against hHb, although remained fully active against a number of generic peptide and protein substrates (Pandey et al., 2005). Additionally, hHb was able to bind to falcipain-2 molecules with the active site blocked by the irreversible inhibitor E-64, clearly indicating that the recognition of intact hHb is mostly independent of the active site. Considering all these observations, we can hypothesize that the binding of odanacatib to falcipain-2 active site would not be likely to perturb substantially the binding affinity of intact hHb to its exosite and *vice versa*. Thus, with the ability to bind both enzyme forms (falcipain-2 and the falcipain-2-hHb complex) with comparable affinity, odanacatib would probably behave as a non-competitive inhibitor against an exosite-binding substrate such as intact hHb. This has been previously reported for small active site-directed inhibitors of proteases and kinases when acting on their natural macromolecular substrates (Krishnaswamy and Betz, 1997; Pedicord et al., 2004; Blat, 2010). The change in the inhibition modality of odanacatib, however, seems insufficient to explain, *per se*, the magnitude of the drop in its inhibitory potency against the falcipain-2/hHb system. A second line of argument came from the observation that hHb molecule is a catalytically heterogeneous substrate, comprising numerous independent falcipain-2 cleavage sites and whose digestion seems to be a non-ordered process (Subramanian et al., 2009). The occurrence of multiple cleavage events at different sites along the protein sequence leads to the formation of numerous digestion products of lower molecular weight. These digestion products also contain functional falcipain-2 cleavage sites and become new substrates that, after a new round of proteolysis, may generate additional substrate peptides. As the reaction proceeds, this iterative process leads to an increase in the number and the global concentration of peptidyl substrates able to compete for the binding to falcipain-2 active site. This might eventually lead to a partial relief of inhibition by competitive, active site-directed inhibitors, as would be the case of odanacatib.

Considering that falcipain-2 and−3 are the major cysteine proteases required for the intraerythrocytic development of *P. falciparum*, we evaluated the antiparasitic effect of odanacatib and methacycline. The inhibitors of cysteine proteases block the hydrolysis of hemoglobin, causing the development of enlarged, hemoglobin-filled food vacuoles in trophozoites and failure of parasites to complete their development (Marco and Coterón, 2012). The two drugs showed a clear inhibition in a dose-depend manner on the intraerythrocytic cycle of *P. falciparum*. The effective concentrations of odanacatib in *P. falciparum* cultures were in the low-to-middle micromolar range, similar to those observed in the inhibition assay of hemoglobinase activity. This finding is compatible with a hypothetical mode of action trough inhibition of falcipain functions within the food vacuole. Although the inhibition of diverse proteases by tetracycline derivatives has previously been reported (Morrison, 1969; Zucker et al., 1985; Sanchez Mejia et al., 2001; Chi et al., 2011), to the best of our knowledge, this is the first report of the inhibition of a C1A cysteine peptidase from a protozoa parasite by an antibiotic of the tetracycline family. The molecular targets for the action of tetracyclines against *Plasmodium* parasites have not been fully elucidated. However, their mode of action seems to include the inhibition of protein synthesis at mitochondrial, plastid and nuclear ribosomes by the association with ribosomal components (Gaillard et al., 2015). Additional mechanisms, such as reduction in *de novo* pyrimidine synthesis and a decrease in the transcription rates of mitochondrial and apicoplast genes, have also been postulated (Briolant et al., 2008; Gaillard et al., 2015). The inclusion of falcipain-2 among the potential targets of tetracycline derivatives adds new possibilities for the development of “two-edged swords” candidate drugs for *P. falciparum*, with potential benefits in terms of potency and delay of resistance appearance (Agarwal et al., 2017). The contribution of this mechanism to the global antimalarial activities of these antibiotics remains to be established in future investigations.

It should also be underlined that odanacatib underwent long-term clinical trials as a treatment of postmenopausal osteoporosis (Bone et al., 2015), which were early stopped due to robust efficacy and a favorable benefit/risk profile. However, its clinical development was dropped due to an increased risk of stroke in the postmenopausal patients on odanacatib vs. a placebo group. Accordingly, our findings on the potential use of the drug against malaria could be considered a drug rescue example, i.e., a proposal on a new medical used of and abandoned or discontinued drug. Do the safety issues of odanacatib pose an inevitable impediment for their potential development as antimalarial treatment? Not necessarily. Although they are indeed a concern, it should be considered that the odanacatib augmented risk of stroke was observed in long-term studies, whereas the drug could possibly be administered in a short-term manner as malaria treatment (for instance, artemisinin-based combination therapies only require a 3-day course to achieve efficacy in cases of uncomplicated *P. falciparum* malaria). Accordingly, the long-term risks of odanacatib use may not have a negative impact on its use as antimalarial. There are well-known examples of drug rescue of discontinued drugs with severe safety issues, that can be re-introduced in a new therapeutic setting with the pertinent precautions. For instance, thalidomide was largely abandoned due to its teratogenic effects, but has been recently relaunched to the market for the treatment of leprosy and multiple myeloma (Teo et al., 2002; Mercurio et al., 2017).

Pharmacokinetics studies reveal that after multiple-dose administration of odanacatib 50 mg (once weekly for 4 weeks), average maximal plasma concentrations of around 400 nM are observed (Chen et al., 2018), although a high fraction of plasma protein bound drug has also been reported (Kassahun et al., 2014). Accordingly, further studies are required to evaluate the dose-compatibility between the previously investigated therapeutic use and the possible antimalarial indication.

## Data Availability

All datasets generated for this study are included in the Supplementary Data Sheet 1.

## Ethics Statement

Human erythrocytes were obtained from volunteer donors with a procedure approved by CEIC (Committee for Ethics on Clinical Investigation, Facultad de Farmacia y Bioquímica, Universidad de Buenos Aires EXP-UBA: 0048676/2017).

## Author Contributions

All authors listed have made a substantial, direct and intellectual contribution to the work, and approved it for publication.

### Conflict of Interest Statement

The authors declare that the research was conducted in the absence of any commercial or financial relationships that could be construed as a potential conflict of interest.
