# Supplementary material for: In silico Guided Drug Repurposing: Discovery of New Competitive and Non-competitive Inhibitors of Falcipain-2
Source: Front Chem. 2019 Aug 6;7:534. doi: 10.3389/fchem.2019.00534 (PMC6691349; doi:10.3389/fchem.2019.00534)
Supplement: Supplementary file 2 [file Data_Sheet_2.docx]

**List of the best eleven individual models in the DUDE-A library**

**Model 594** = -0.48333 + 0.38415***SM08_AEA(bo)** - 6.50601***SpPosA_A** - 0.12786***C-005** + 0.35800***B05[N-N]** + 0.13459***nR=Cs** + 0.21576***CATS2D_02_DD** + 0.25881***nS(=O)2** - 0.33510***B03[O-S]** - 0.07816***N-072** + 0.16832***B06[C-S]**

**Model 516** = 2.15028 + 0.51394***B01[O-S]** - 1.39090***SpMAD_B(v)** + 1.28453***MATS1i** + 0.78666***B01[O-O]** + 0.10578***nR=Cs** + 0.30657***F10[N-N]** + 0.01297***EE_D/Dt** - 0.05929***O-060** - 0.12593***F04[N-N]** + 0.18056* **B06[N-Cl]**

**Model 477** = -0.20183 - 0.11308***SddssS** + 0.11304***C-003** + 3.08845***MATS1v** + 0.03315***PHI** + 0.28521***nC=N-N<** + 0.09734***nR=Cs** + 0.23815***F10[N-N]**

**Model 975** = -1.31004 + 0.91435***SM03_EA** - 3.71481***Eta_betaS_A** + 0.39261***nCONN** + 0.52812***B10[N-S]** + 0.10146***O-057** - 0.02521***F07[C-O]** - 0.07950***H-051** + 0.17105***H-048** + 0.30706***C-038** - 0.80895***MATS2i** + 0.21603***J_D**

**Model 244** = -0.113068 + 0.994507***MATS2s** + 0.414362* **MATS7e** + 0.138526***nR=Cs** + 0.536027***B10[N-S]** + 1.267066***MATS1i** + 0.825085***B01[O-O]** + 0.317380***nCONN** + 0.002292***ATSC5s** + 0.304093***B10[N-N]** - 0.159143***MPC07** + 0.213534***Eig02_AEA(dm)**

**Model 504** = 6.7518 - 7.5560***AVS_X** + 0.4767***B01[O-S]** + 0.1801***nC=N-N<** + 0.8503***GATS2p** + 0.1194***nR=Cs** + 0.8342***B01[O-O]** + 0.4394***CATS2D_02_DD** - 0.6738***GATS3e** + 0.1725***Eig05_EA(bo)**

**Model 870** = -0.04303 - 0.12937***SddssS** + 0.73875***F01[O-O]** + 0.12965***H-050** + 0.06528***SdsCH** + 0.15711***F05[C-Cl]** - 0.30359***F04[N-Cl]** + 0.15323***B04[O-O]** + 0.72561***MATS4v** -0.05329***F06[O-O]**

**Model 154** = -0.062743 + 0.053532***GGI1** + 0.455998***B01[O-S]** + 0.166891***Hy** + 0.667469***B01[O-O]** - 0.072915***F09[O-O]** - 0.733720***MATS5m** + 0.212851***NsCl** - 0.121481***F04[N-N]** -0.004399***T(O..Cl)**

**Model 764** = -0.492730 + 0.065886***CATS2D_04_DL** + 0.603836***S-110** + 0.233321***nR11** -0.374767***F01[N-S]** + 0.308977***B08[N-Cl]** + 0.003437***TPSA(Tot)** - 0.071322***F10[O-O]** + 0.141029***J_Dz(e)**

**Model 564** = 11.77564 + 0.05363***X2sol** - 12.56366***AVS_X** - 0.73252***Eig08_EA(dm)** + 0.18653***NdsCH** + 1.55032***MATS1p** + 0.27091***nS(=O)2** + 0.34985***F10[N-N]** + 0.76181* **F01[O-O]** - 0.34692***B04[N-N]** - 0.01890***F02[C-O]**

**Model 80** = -1.83171 + 0.06675***CATS2D_04_DL** + 0.62284***F07[N-S]** + 0.46695***IVDE** + 0.78795***F01[O-O]** + 0.27361***B03[N-S]** + 0.48775***Psi_i_A** + 0.27306***B09[N-Cl]**

**Brief description of the included molecular descriptors in the models.**

| Molecular descriptor | Description |
| --- | --- |
| ATSC5s | Centred Broto-Moreau autocorrelation of lag 5 weighted by I-state |
| AVS_X | Average vertex sum from chi matrix |
| B01[O-O] | Presence/absence of O - O at topological distance 1 |
| B01[O-S] | Presence/absence of O - S at topological distance 1 |
| B03 [O-S] | Presence/absence of O - S at topological distance 3 |
| B03[N-S] | Presence/absence of N - S at topological distance 3 |
| B04[N-N] | Presence/absence of N - N at topological distance 4 |
| B04[O-O] | Presence/absence of O - O at topological distance 4 |
| B05[N-N] | Presence/absence of N - N at topological distance 5 |
| B06[C-S] | Presence/absence of C - S at topological distance 6 |
| B06[N-Cl] | Presence/absence of N - Cl at topological distance 6 |
| B08[N-Cl] | Presence/absence of N - Cl at topological distance 8 |
| B09[N-Cl] | Presence/absence of N - Cl at topological distance 9 |
| B10[N-N] | Presence/absence of N - N at topological distance 10 |
| B10[N-S] | Presence/absence of N - S at topological distance 10 |
| C-003 | CHR3 |
| C-005 | CH3X |
| C-038 | Al-C(=X)-Al |
| CATS2D_02_DD | CATS2D Donor-Donor at lag 02 |
| CATS2D_04_DL | CATS2D Donor-Lipophilic at lag 04 |
| CATS2D_04_DL | CATS2D Donor-Lipophilic at lag 04 |
| EE_D/Dt | Estrada-like index (log function) from distance/detour matrix |
| Eig02_AEA(dm) | Eigenvalue n. 2 from augmented edge adjacency mat. weighted by dipole moment |
| Eig05_EA(bo) | Eigenvalue n. 5 from edge adjacency mat. weighted by bond order |
| Eig08_EA(dm) | Eigenvalue n. 8 from edge adjacency mat. weighted by dipole moment |
| Eta_betaS_A | Eta sigma average VEM coun |
| F01[N-S] | Frequency of N - S at topological distance 1 |
| F01[O-O] | Frequency of O - O at topological distance 1 |
| F02[C-O] | Frequency of C - O at topological distance 2 |
| F04[N-N] | Frequency of N - N at topological distance 4 |
| F04[N-N] | Frequency of N - N at topological distance 4 |
| F05[C-Cl] | Frequency of C - Cl at topological distance 5 |
| F06[O-O] | Frequency of O - O at topological distance 6 |
| F07[C-O] | Frequency of C - O at topological distance 7 |
| F07[N-S] | Frequency of N - S at topological distance 7 |
| F09[O-O] | Frequency of O - O at topological distance 9 |
| F10[N-N] | Frequency of N - N at topological distance 10 |
| F10[N-N] | Frequency of N - N at topological distance 10 |
| F10[O-O] | Frequency of O - O at topological distance 10 |
| GATS2p | Geary autocorrelation of lag 2 weighted by polarizability |
| GATS3e | Geary autocorrelation of lag 3 weighted by Sanderson electronegativity |
| GGI1 | Topological charge index of order 1 |
| H-048 | H attached to C2(sp3)/C1(sp2)/C0(sp) |
| H-050 | H attached to heteroatom |
| H-051 | H attached to alpha-C |
| Hy | Hydrophilic factor |
| IVDE | Mean information content on the vertex degree equality |
| J_D | Balaban-like index from topological distance matrix (Balaban distance connectivity index) |
| J_Dz(e) | Balaban-like index from Barysz matrix weighted by Sanderson electronegativity |
| MATS1i | Moran autocorrelation of lag 1 weighted by ionization potential |
| MATS1p | Moran autocorrelation of lag 1 weighted by polarizability |
| MATS1v | Moran autocorrelation of lag 1 weighted by van der Waals volume |
| MATS2i | Moran autocorrelation of lag 2 weighted by ionization potential |
| MATS2s | Moran autocorrelation of lag 2 weighted by I-state |
| MATS4v | Moran autocorrelation of lag 4 weighted by van der Waals volume |
| MATS5m | Moran autocorrelation of lag 5 weighted by mass |
| MATS7e | Moran autocorrelation of lag 7 weighted by Sanderson electronegativity |
| MPC07 | Molecular path count of order 7 |
| N-072 | RCO-N< / >N-X=X |
| nC=N-N< | Number of hydrazones |
| nCONN | Number of urea (-thio) derivatives |
| NdsCH | Number of atoms of type dsCH |
| nR=Cs | Number of aliphatic secondary C(sp2) |
| nR11 | Number of 11-membered rings |
| nS(=O)2 | Number of sulfones |
| NsCl | Number of atoms of type sCl |
| O-057 | Phenol / enol / carboxyl OH |
| O-060 | Al-O-Ar / Ar-O-Ar / R..O..R / R-O-C=X |
| PHI | Kier flexibility index |
| Psi_i_A | Intrinsic state pseudoconnectivity index - type S average |
| S-110 | R-SO2-R |
| SddssS | Sum of ddssS E-states |
| SdsCH | Sum of dsCH E-states |
| SM03_EA | Spectral moment of order 3 from edge adjacency mat. |
| SM08_AEA(bo) | Spectral moment of order 8 from augmented edge adjacency mat. weighted by bond order |
| SpMAD_B(v) | Spectral mean absolute deviation from Burden matrix weighted by van der Waals volume |
| SpPosA_A | Normalized spectral positive sum from adjacency matrix |
| T(O..Cl) | Sum of topological distances between O..Cl |
| TPSA(Tot) | Topological polar surface area using N,O,S,P polar contributions |
| X2sol | solvation connectivity index of order 2 |
