# Supplementary material for: In silico Guided Drug Repurposing: Discovery of New Competitive and Non-competitive Inhibitors of Falcipain-2
Source: Front Chem. 2019 Aug 6;7:534. doi: 10.3389/fchem.2019.00534 (PMC6691349; doi:10.3389/fchem.2019.00534)
Supplement: Supplementary file 3 [file Data_Sheet_3.pdf]

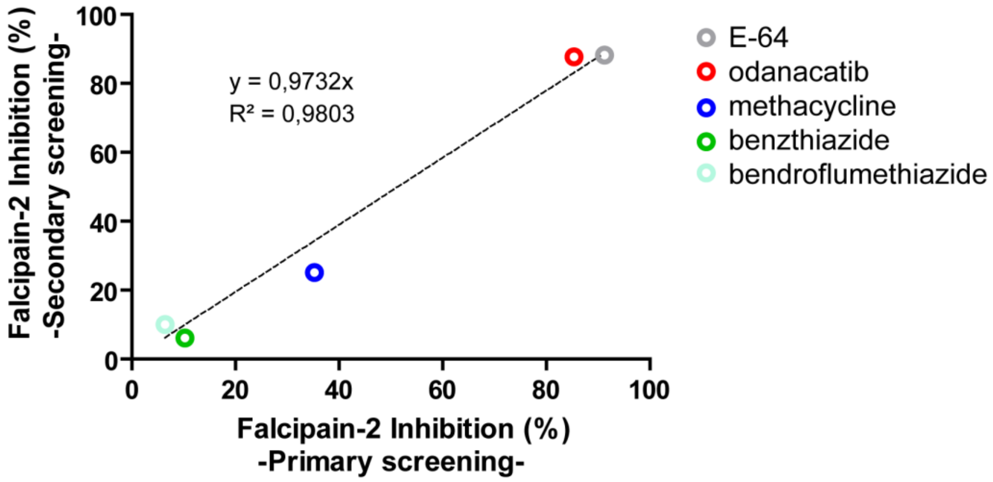

Supplementary Figure 1: Correlation between inhibition percentages in the primary and secondary screenings for investigational compounds. Analysis was performed using a single concentration of 31.25 µM and 23.4 µM for primary and secondary screening, respectively.

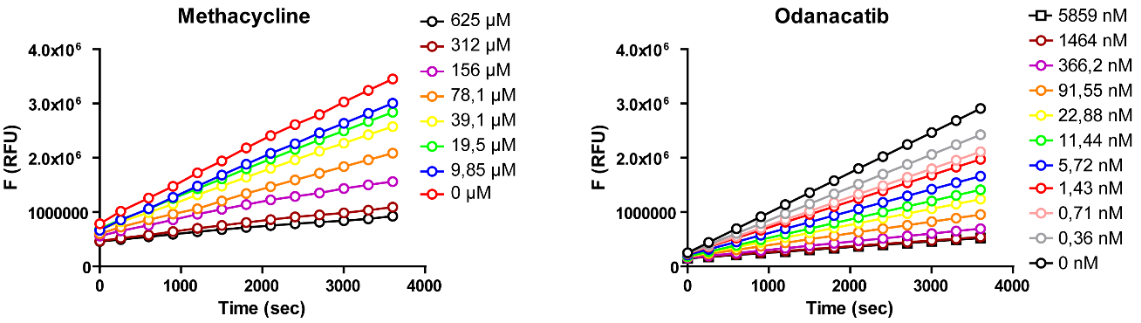

Supplementary Figure 2: Collection of progression curves for the most active compounds. **(A)** Methacycline. **(B)** Odanacatib. Each curve corresponds to a different compound concentration, as indicated. From the 24 concentrations tested for each compound, only a few curves evenly distributed among the complete inhibition range, were selected for representation.

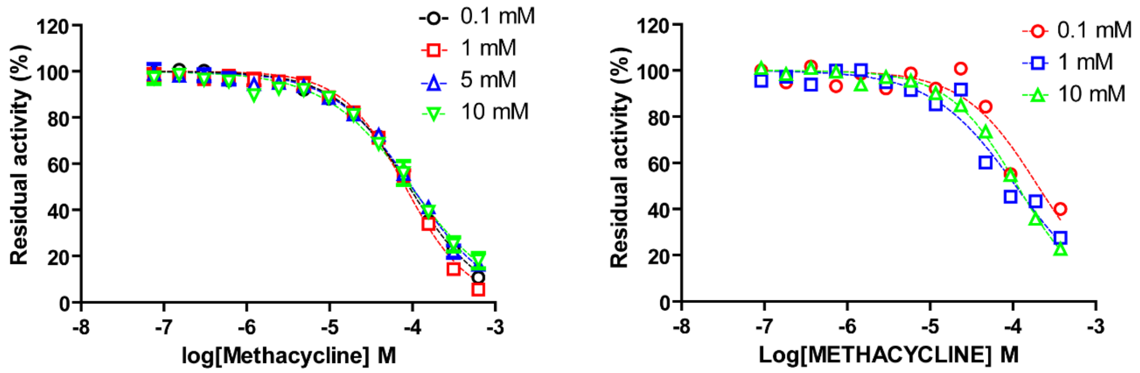

Supplementary Figure 3. Inhibition of falcipain-2 by methacycline is insensitive to the strength and concentration of reducing agents. **(A)** Dose-response curves for methacycline at fixed concentrations of DTT, a strong reducing agent. **(B)** Dose-response curves for methacycline at fixed concentrations of cysteine, a weak reducing agent. In all cases, dotted lines represent the best fit of experimental data to the four-parameter Hill equation.

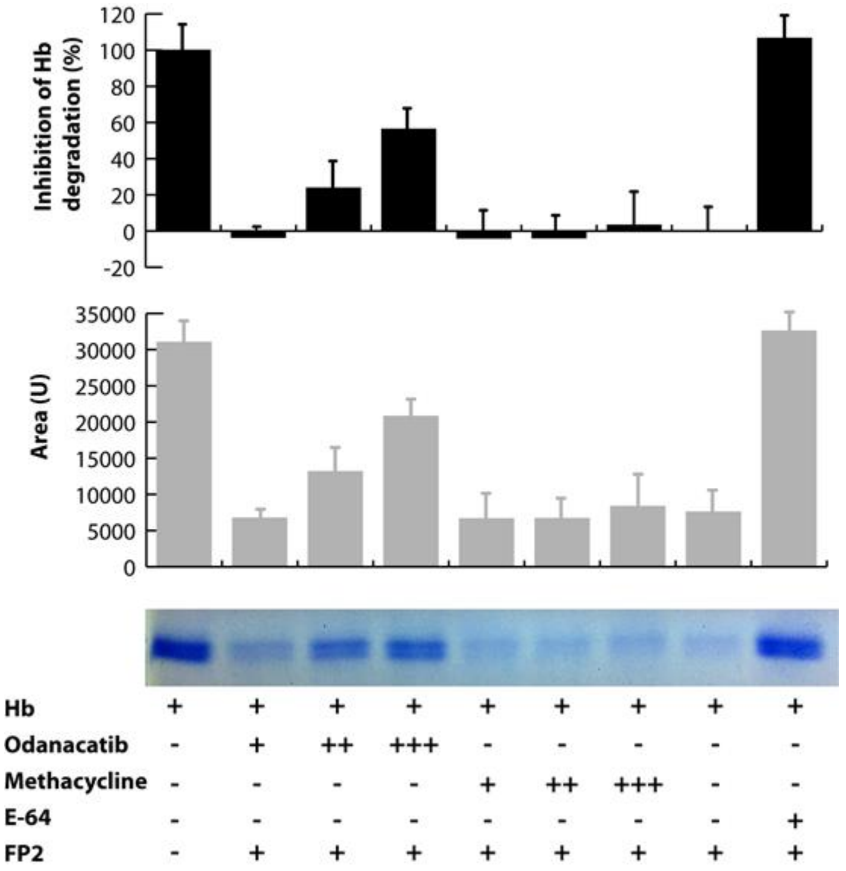

Supplementary Figure 4. Inhibition of falcipain-2 hemoglobinase activity by odanacatib and methacycline. Bottom panel shows Coomassie blue stained SDS-PAGE (representative experiment) and summarizes the composition of individual reaction mixes. Central panel (grey bars) shows direct densitometric quantification of hHb bands. Data correspond to three independent experiments. The corresponding inhibition percentages are indicated in the top panel (black bars).

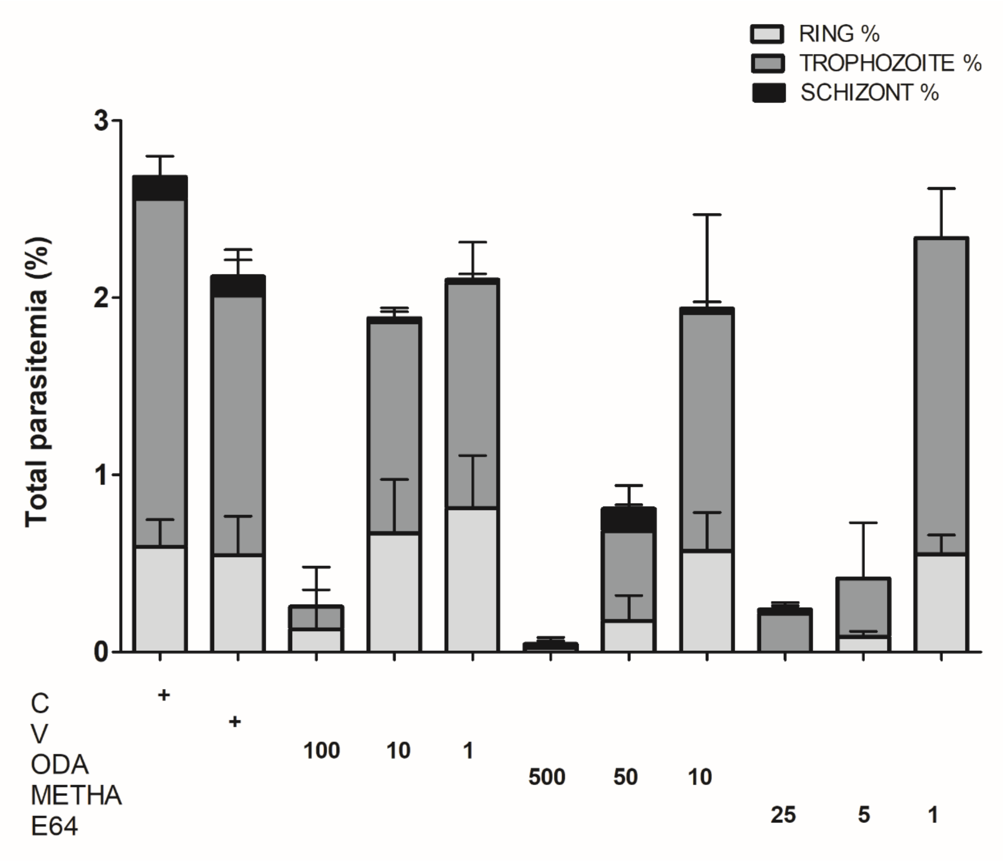

Supplementary Figure 5: Effect of odanacatib, methacycline and E-64 on the development of *P. falciparum* under culture by parasite-stage. Cultures of erythrocytes infected (trophozoite stage) at 2% hematocrit and 0.5% parasitemia were incubated with increasing concentrations of odanacatib (ODA; 1, 10 or 100 µM), methacycline (METHA; 10, 50 or 500 µM) and E-64 (1, 5 or 25 µM). DMSO was used as a vehicle control (V) and RPMI 1640 medium as a control (C). After 48 h, the number of infected erythrocytes at each intraerythrocytic stage (ring, trophozoite or schizont-stage) was evaluated by light microscopy in stained blood smears. Parasitemia was calculated as (infected erythrocytes / total erythrocytes)*100 for each stage. Total parasitemia derives from the sum of the three stages. Data are the means ± SD of one experiment performed by triplicate.

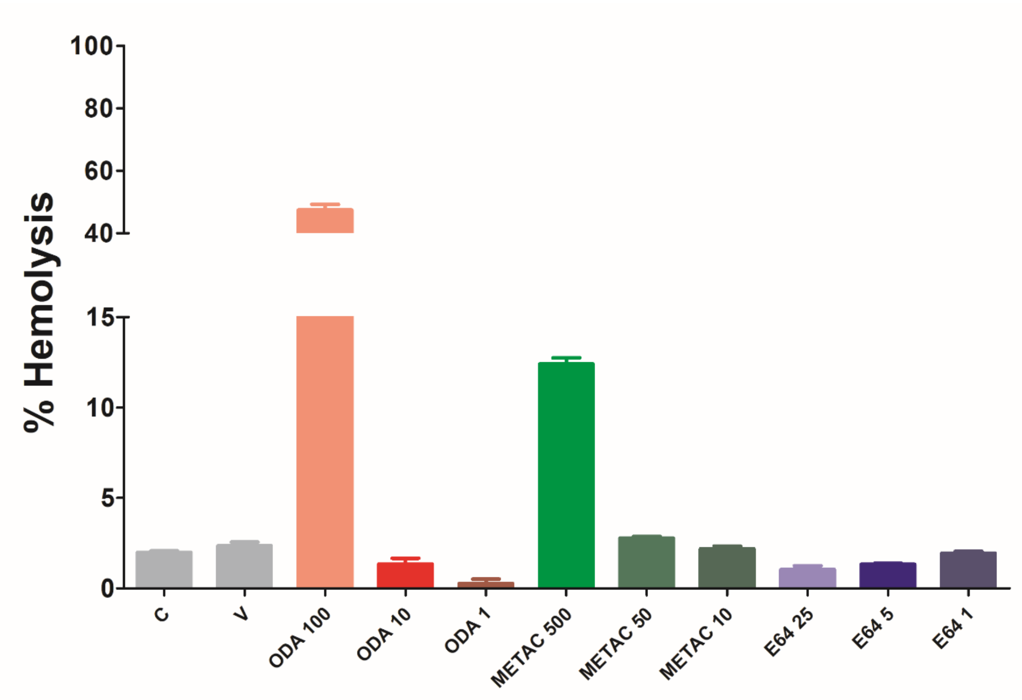

Supplementary Figure 6: Cytotoxic effect of odanacatib, methacycline and E-64 on the development of *P. falciparum* under culture. One hundred µl of synchronous trophozoite-stage infected erythrocytes cultures were plated in 96-well at 4 % hematocrit and 1 % parasitemia. One hundred µL of odanacatib (ODA; 200, 20 or 2 µM), methacycline (METHA; 1000, 100 or 20 µM), E-64 (50, 10 or 2 µM), DMSO (vehicle control; V) or RPMI 1640 medium (control; C) were dispensed into each well to achieve final hematocrit of 2%, 0.5% parasitemia and the final concentration of each compound tested, in a final volume of 200 µl. After 48 h cultivation at 37 °C, the plate was centrifuged (600 x g at 20 °C for 3 min) and the hemolysis was quantified as released hHb by measuring absorbance at 405 nm on the supernatant. Total lysis was prepared from a control treatment completely lysed with distilled water. A calibration curve was done by 2-serial dilutions and Absorbance vs % of hemolysis was fitted to a linear function. Results are expressed as percentage of hemolyzed erythrocytes (% Hemolysis). Data are the means ± SD of one experiment performed by triplicate.
